# Student Attainment of Proficiency in a Clinical Skill: The Assessment of Individual Learning Curves

**DOI:** 10.1371/journal.pone.0088526

**Published:** 2014-02-20

**Authors:** Robert D. Campbell, Kent G. Hecker, David J. Biau, Daniel S. J. Pang

**Affiliations:** 1 Calgary Animal Referral and Emergency Centre, Calgary, Alberta and the Faculty of Veterinary Medicine, University of Calgary, Calgary, Alberta, Canada; 2 Veterinary Clinical and Diagnostic Sciences, Faculty of Veterinary Medicine, University of Calgary, Calgary, Alberta, Canada; 3 Département de Biostatistique et Informatique Médicale, Hôpital Saint-Louis, AP-HP Paris, France; 4 Hotchkiss Brain Institute, University of Calgary, Calgary, Alberta, Canada; World Health Organization, Switzerland

## Abstract

The aims of this study were to determine if the learning curve cumulative summation test (LC-CUSUM) can differentiate proficiency in placing intravenous catheters by novice learners, and identify the cause of failure when it occurred. In a prospective, observational study design 6 undergraduate students with no previous experience of placing intravenous catheters received standardized training by a board certified veterinary anesthesiologist in intravenous catheter placement technique. Immediately following training, each student attempted 60 intravenous catheterizations in a dog mannequin thoracic limb model. Results were scored as a success or failure based upon completion of four specific criteria, and where catheter placement failure occurred, the cause was recorded according to pre-defined criteria. Initial acceptable and unacceptable failure rates were set by the study team and the LC-CUSUM was used to generate a learning curve for each student. Using 10% and 25% acceptable and unacceptable failure rates, 3 out of 6 students attained proficiency, requiring between 26 to 48 attempts. Applying 25% and 50% acceptable and unacceptable failure rates, 5 of 6 students obtained proficiency, requiring between 18 and 55 attempts. Wide inter-individual variability was observed and the majority of failed catheterisation attempts were limited to two of the four pre-defined criteria. These data indicate that the LC-CUSUM can be used to generate individual learning curves, inter-individual variability in catheter placement ability is wide, and that specific steps in catheter placement are responsible for the majority of failures. These findings may have profound implications for how we teach and assess technical skills.

## Introduction

Attainment of proficiency in a technical skill, such as intravenous (IV) catheterization, is often based on an arbitrary distinction such as experience, or instructor observation of one or more successful attempts. Evidence suggests there is wide variation between individual attainment of proficiency in a technical skill. [1], [2] The concept of quantitatively assessing proficiency in clinical skills for individual students is relatively new to the health professions and has garnered recent prominence in human medicine as a result infamous cases such as increased mortality rates following cardiac surgery [3]–[5].

Statistical process control charts, originally designed for use in the manufacturing industry to assess production processes have recently been adapted to quantitatively assess attainment and maintenance of proficiency in medicine, particularly in surgery and anesthesia. [1], [6], [7] The cumulative summation (CUSUM) method tests the hypothesis that a process (e.g. surgical performance in cardiac surgery) is deviating from a target of adequate performance (process is out of control), or that a process remains within an acceptable limit (process is in control). [8] If a process is deemed to be ‘out of control’, an intervention takes place, such as a period of re-training. The limitation of this technique during an initial period of learning is that the learner will be frequently assessed as out of control due to poor performance, triggering an unacceptable failure rate. Biau and Porcher modified the CUSUM test to provide monitoring and assessment of when a process reaches an in control state, creating the LC-CUSUM (the cumulative summation test for learning curve). This approach considers that a process is out of control initially (as a result of initial trainee performance), and indicates when the process is in control (adequate trainee performance achieved). [9].

Training and assessing veterinary students to an acceptable level of proficiency in clinical skills, such as venous catheterization, and decisions regarding exposure to practical classes (with mannequins, simulators or live animals) should be based on evidence. This is particularly important as the use of in vivo models for teaching purposes must be defensible, in keeping with the principles of the 3 Rs (replacement, reduction, refinement) of animal use. [10], [11] Furthering our understanding of the learning curve would allow an optimal use of training resources, thereby reducing associated costs. To use IV catheterization as an example, the choice of training tool (live animal versus model) could be guided by the principle of the right tool at the right time: a low fidelity model may be adequate for understanding the basic steps of IV catheter placement while a high fidelity simulator or live animals may only be necessary when attempting to create a clinical scenario.

For the first time in veterinary medicine the LC-CUSUM test was applied to evaluate whether proficiency in placing intravenous (IV) catheters could be achieved by subjects with no previous experience with this skill.

## Materials and Methods

This research project received ethics approval from the University of Calgary Conjoint Health Research Ethics Board (E-24326). All participants provided informed signed consent.

Six undergraduate students (from the Bachelor of Health Sciences program at the University of Calgary) participated in the study. Exclusion criteria included previous experience observing or performing IV catheter placement in any species. Students were required to perform 60 IV catheter attempts in a dog forelimb mannequin (K9 IV Trainer, Rescue Critters, Van Nuys, CA, USA). The artificial veins were filled with artificial blood maintained at a pressure of approximately 100 mmHg. This was achieved by placing the fluid bag in an inflatable pressure sac. All students used the same model of IV catheter (PROTECTIV ® 20G 1.25″, Smiths Medical, Markham, ON, Canada). Prior to proceeding with the experiment students were instructed individually by a board- certified anesthesiologist (DP) using a standardized series of instructions. Testing was video-recorded to allow for retrospective analysis and ensure uniformity of grading between examiners. Students were assigned a binary score for each attempt (0 =  failure, 1 = success), which were used to generate a learning curve. The scorers did not provide students with any feedback during the simulations.

**Figure 1:**
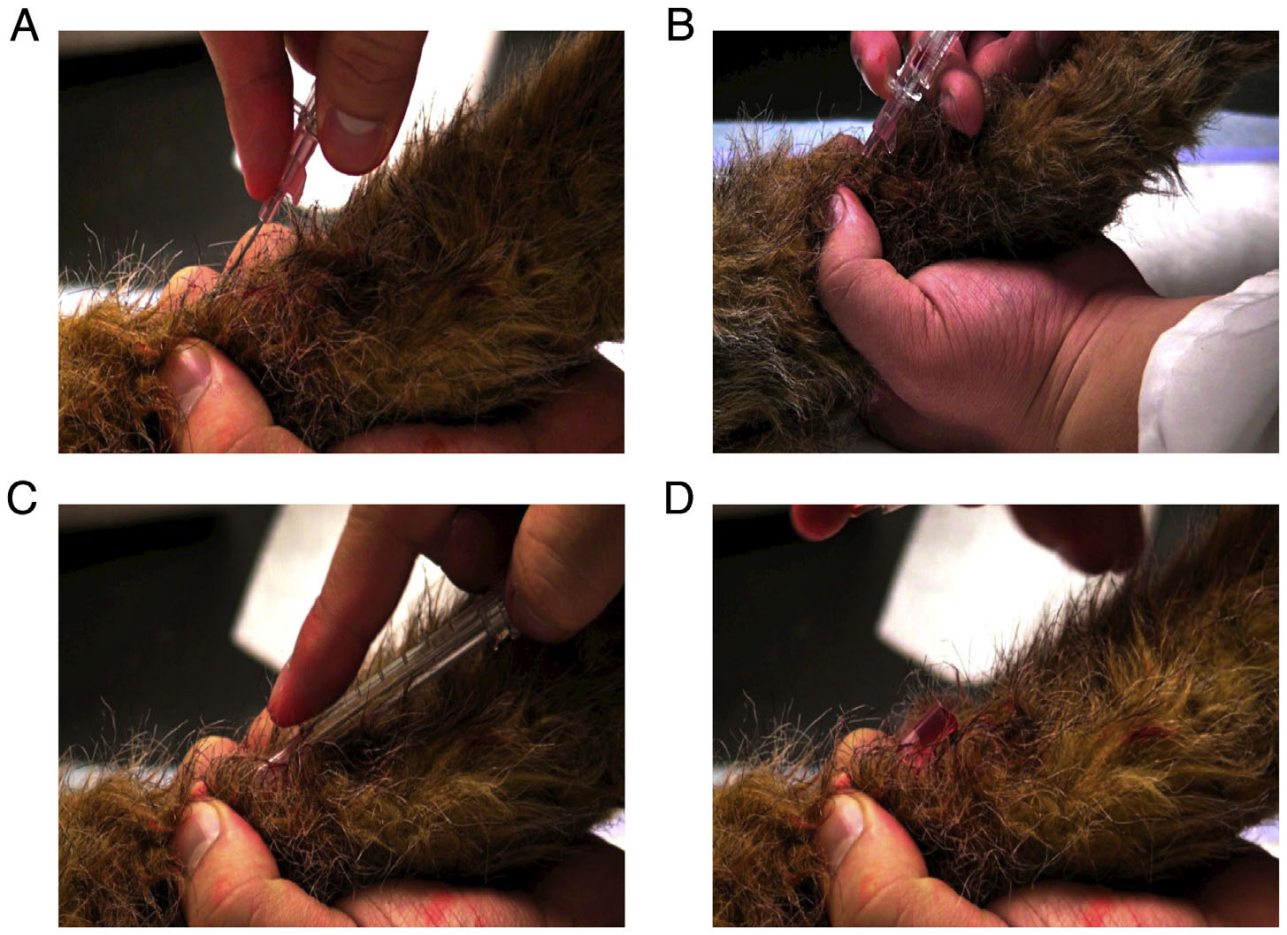
Video stills showing key steps in the successful catheter placement being implemented in the canine IV training model. A: Piercing the skin with the catheter and stylet at a 20–40 degree angle (relative to long axis of mannequin limb) B: Advancing the catheter and stylet into the vein (confirmed by observation of a “flash” of blood in the catheter hub). C: Reducing the angle of the catheter and stylet and advancing them 1–2 mm further to ensure placement in the vein before advancing the catheter in to the vein while holding the stylet in place. D: Observing blood flow from the catheter hub.

To be deemed successful each student was required to (Figure 1):

1. Pierce the skin with the catheter and stylet at a 20–40 degree angle (relative to long axis of mannequin limb)
2. Advance the catheter and stylet into the vein (confirmed by observation of a “flash” of blood in the catheter hub).
3. Reduce the angle of the catheter and stylet and advance them 1–2 mm further to ensure placement in the vein before advancing the catheter in to the vein while holding the stylet in place.
4. Observe blood flow from the catheter hub

Participants who did not complete all of these components were scored as a failure. An additional sub-score was recorded reflecting at which point of the 4 steps failed catheterization attempts occurred. Students were not provided a time limit for the simulation, but were required to take a 1-minute break after every 3 attempts.

The LC-CUSUM sequentially tests, after each procedure, the null hypothesis that performance is inadequate. A score is computed from successive procedures with successes yielding an increase in the score and failures yielding a decrease. [9] Once the score reaches a predefined value (h, the in-control limit), the null hypothesis is rejected and performance is considered adequate. The in-control limit, which defines when proficiency is achieved, is based on numerical simulations of the probability of early detection of proficiency (see Marshall et al. and Biau and Porcher for statistical review). [9], [12] Graphically, the LC-CUSUM score is displayed on the y-axis and procedure number on the x-axis. Performance of the trainee is considered to be unacceptable as long as the score remains below the in-control limit, h. The trainee is considered proficient when the in-control limit line is reached. A holding barrier at y = 0 ensures that a student’s learning curve will not suffer unduly (become negative) for poor previous performance, thereby allowing the test to remain responsive to present performance.

Defining adequate and inadequate performance should be based on expert consensus or existing specialty college guidelines; such guidelines do not exist in veterinary medicine. Because these parameters control the weighting allocated to failure or success at a task, adequate and inadequate performance should be set according to context i.e. the level of experience/training of the population being studied. In this study the acceptable and unacceptable failure rates were initially assigned after consultation with a board certified anesthesiologist (DP) with experience of training undergraduate and postgraduate trainees.

For the present study, the adequate performance level was initially set at 10% failure, inadequate performance at 25% failure, and the acceptable deviation from adequate performance was 5%. An in-control limit, h = 0.95, was chosen to give a true discovery rate (probability of declaring competency if the trainee’s true performance is adequate; true alarm, akin to power) of 87% and a false discovery rate (probability of declaring competency if the trainee’s false performance is inadequate; false alarm, akin to type I error) of 14% over 60 procedures. Adequate and inadequate performance, and acceptable deviation criteria were then adjusted to be more lenient (25%, 50%, and 10% respectively) to assess when (or if) more students achieved proficiency. In this case, a limit h = 1.15 was chosen to give a true discovery rate of 94% and a false discovery rate of 10% over 60 procedures.

## Results

**Figure 2:**
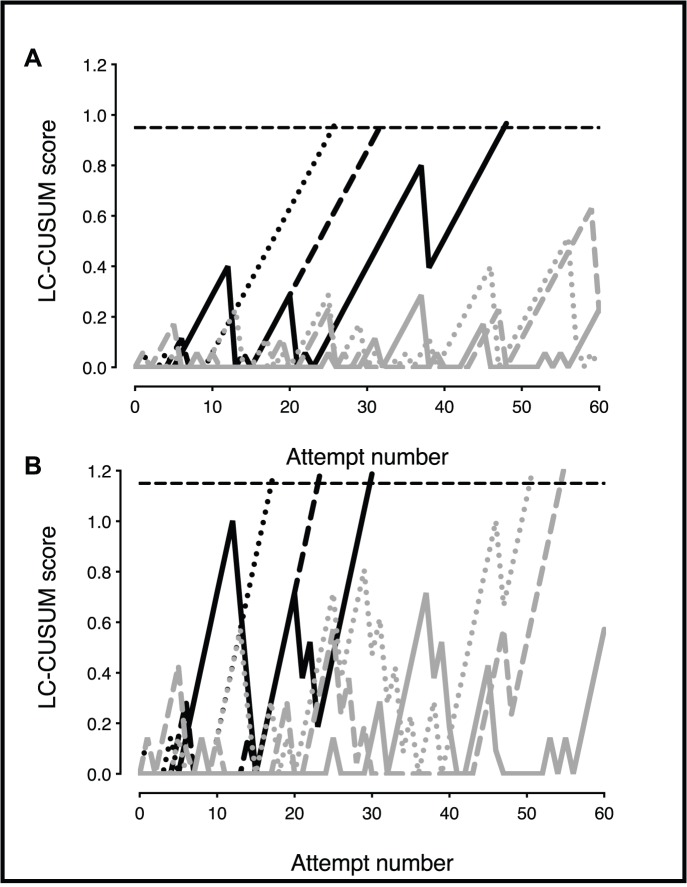
A, LC-CUSUM plot. Horizontal dashed line represents the in-control limit, the threshold for achieving proficiency. Lines represent individual student learning curves. Three students achieved proficiency within 60 attempts as depicted by transection of the in-control limit. Two students did not. Acceptable and unacceptable failure rates were set at 10% and 25%, respectively. N = 6 students. B, Same raw data as in 2A, but acceptable and unacceptable failure rates were set at 25% and 50%, respectively. This change in performance levels results in students attaining proficiency sooner, and two additional students attain proficiency. The in-control limit (horizontal dashed line) changes with a change in performance levels in order to control Type 1 and II errors. Student 1 (—), student 2 (---), student 3 (---), student 4 (….), student 5 (—), student 6 (….).

Figure 2 presents the results comparing student proficiency based upon the two sets of acceptable and unacceptable failure rates. With acceptable and unacceptable failure rates of 10 and 25% (Figure 2A) respectively, 3 out of 6 students achieved proficiency. The number of attempts required to achieve proficiency (reach the in-control limit line) were 26, 32 and 48. Three students did not achieve proficiency within 60 attempts. Lowering the standard for acceptable and unacceptable failure rates to 25 and 50% respectively resulted in 5 of 6 students attaining proficiency (Figure 2B). Considerable inter-individual variability was reflected in the number of attempts required to attain proficiency, varying from 18 to 55 (Figure 2B).

Examination of the point of failure for individual students (Table 1), reveals that the overwhelming majority (95.7%) of failed catheterizations occurred during steps 2 and 3 of the 4-step procedure. Of these, 57 (39.8%) were accounted for by step 2, and 80 (55.9%) accounted for by step 3.

**Table 1:** Total number of unsuccessful catheter placement attempts per student, categorised according to observable criteria.

Criteria	Student 1	Student 2	Student 3	Student 4	Student 5	Student 6
Pierce skin withcatheter at 20–40°	0	0	0	1	5	0
Advance catheter and stylet –observe flash of blood	0	6	3	17	24	7
Thread catheter off stylet	10	15	29	4	12	10
Observe blood flow	0	0	0	0	0	0

See text for full description of criteria.

## Discussion

Clinical proficiency has historically been determined by utilizing subjective criteria; for example, after an arbitrary number of procedural attempts, or after a certain amount of time has passed with assumed proficiency (Rush et al, 2011). [13] Assuming proficiency based on generalized criteria does not take into account inter-individual variability in learning, nor does it provide for continued assessment of maintained proficiency over time. Recent research in human anesthesia has shown that achieving clinical proficiency varies widely from individual to individual (de Oliveira Filho, 2002; Naik et al, 2003; Konrad et al, 1999). [1], [2], [14] de Oliveira Filho (2002) reported successful endotracheal intubation ranges from 9 to 88 (43±33.49, mean ± SD) attempts in novice anesthesia residents. [1] Another study reported wide individual variation in first year anesthesia residents achieving clinical proficiency in epidural anesthesia (ranging from 1 to 85 attempts, with one resident failing to achieve proficiency after 75 attempts). [2] We have identified large inter-individual variation in achieving proficiency in IV catheter placement, demonstrating the need for increased scrutiny in candidate performance in veterinary medicine, and reform of current training paradigms.

The LC-CUSUM has been previously employed successfully to evaluate achievement of clinical proficiency in human medicine. [15], [16] Definition of acceptable and unacceptable failure rates are crucial to successful utilization of LC-CUSUM, and are typically set by experts in a given field (for example; board certified anesthesiologists for anesthesia procedures). Despite the use of expert consensus there is potential for bias when setting these values, and if criteria are set too strictly achievement of proficiency may be delayed (or in some cases, never achieved by an individual), hence the importance of establishing success and failure rates appropriate to the candidates’ level of training. Ideally bias is decreased by setting acceptable and unacceptable failure rates according to empirical data rather than opinion; empirical training data does not currently exist in veterinary medicine. In this study, when stricter acceptable and unacceptable failure rates were set (10% and 25% respectively, and acceptable deviation from adequate performance of 5%) 3 out of the 6 students failed to achieve proficiency over 60 attempts, and of those who did achieve proficiency it took between 26 and 48 attempts. Changing the threshold for proficiency (changing the acceptable and unacceptable failure rates to 25% and 50% respectively, and acceptable deviation to 10%) affects the number of students attaining proficiency as expected, however wide inter-individual variability remained. These data indicate that traditional methods for assessing proficiency, typically after an arbitrary number of attempts, or time in training, may not adequately cater for individual variation in learning.

These data provide two important pieces of information, the steps associated with impeding the learning curve in a group of naïve students, and the benefit of tracking performance at an individual level.

Examination of the causes of failed catheter placements (Figures 1 and 2), there are multiple sources: 1. Each step of catheter placement must be completed successfully to allow progression to the next step. For example, completion of step 3 is necessary to advance to step 4. There were no failures occurring at step 4 (Table 1), indicating that the critical steps occur earlier in the sequence. Furthermore this indicates that while evaluation of step 4 alone would reflect successful catheter placement, it will not allow identification of the source of failed attempts. In this study, failed attempts occurred primarily at steps 2 and 3. 2. There were a low number (6) of attempts failing at step 1, indicating that this is not a critical step though it is possible that the orientation of the catheter with respect to the long axis of the vein contributed to failures at step 2. Catheter orientation was not evaluated in this study. 3. Additional factors potentially contributing to failure at step 2 include the speed of catheter and stylet advancement and the ability of candidates to identify penetration of the vein from the sensation of advancing the catheter. 4. Successful completion of step 3 requires three distinct steps (reducing the angle of insertion, advancing the catheter and stylet a 1–2 mm further, and threading the catheter in to the vein) which may explain the greater failure rate. 5. Achievement of proficiency (attaining the in-control limit line) is reflected not simply by having a lesser number of total failure rates, but also the number of successive failures. Taking the student represented by the solid black line as an example (Figure 2A), the student approaches the in-control limit following a run of successful catheter placements. However, one failed attempt results in a descent of the learning curve towards zero, requiring a further run of 10 consecutive successes to achieve proficiency.

The performance tracking of individual students allows identification of areas needing further training or practice, or both. For example, student 4 (Table 1) is relatively successful in step 3, with only 4 unsuccessful attempts as a result of failure to thread the catheter in to the vein, but recorded the second highest failure rate at step 2. This contrasts with student 3, where the opposite is apparent, with the second lowest incidence of failures at step 2, but the highest number of failures at step 3. This information could be used to supported targeted training, allowing students and trainers to focus on key areas.

Our results indicate that LC-CUSUM can be used to generate individual learning curves for a technical skill in veterinary medicine and the technique is simple to implement, providing an individualized approach to learning. The results provide further evidence of performance variation by student within task reinforcing the need for learner specific feedback in order to achieve proficiency. In other words, the LC-CUSUM provides instructors and learners specific information which can be used to complement more traditional evaluations of skill performance, such as number of times skill has been performed and duration of time on task. Once students are deemed proficient, CUSUM can be applied to analyze the maintenance of proficiency over time. By increasing the number of students in the experiment, we hope to generate individualized learning curves that can be trended and extrapolated to populations of students to better design teaching methodologies. LC-CUSUM provides data (rather then speculation) that may facilitate guidelines on acceptable levels of proficiency for students at different levels of training and application of the LC-CUSUM derived data may allow for evaluation of teaching techniques (computer simulated learning vs. lectures vs. mannequin models vs live models etc.) in terms of their educational merit in contributing to skilled practitioners.
